# 4-Nitro­aniline–picric acid (2/1)

**DOI:** 10.1107/S1600536809037416

**Published:** 2009-09-30

**Authors:** Yan-jun Li

**Affiliations:** a College of Chemical Engineering and Technology, Wuhan University of Science and Technology, Wuhan 430081, People’s Republic of China

## Abstract

In the title adduct, C_6_H_3_N_3_O_7_·0.5C_6_H_6_N_2_O_2_, the complete 4-nitro­aniline mol­ecule is  generated by a crystallographic twofold axis with two C atoms and two N atoms lying on the axis. The mol­ecular components are linked into two dimensional corrugated layers running parallel to the (001) plane by a combination of inter­molecular N—H⋯O and C—H⋯O hydrogen bonds. The phenolic oxygen and two sets of nitro oxygen atoms in the picric acid were found to be disordered with occupancies of 0.81 (2):0.19 (2) and 0.55 (3):0.45 (3) and 0.77 (4):0.23 (4), respectively.

## Related literature

For background to picrate derivatives, see: Harrison *et al.* (2007 ▶); Pascard *et al.* (1982 ▶); Pearson *et al.* (2007 ▶); Wang *et al.* (2003 ▶).
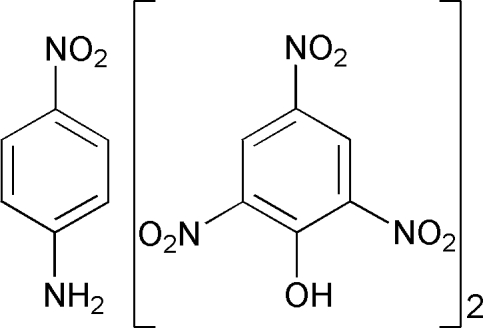


## Experimental

### 

#### Crystal data

C_6_H_3_N_3_O_7_·0.5C_6_H_6_N_2_O_2_
*M* *_r_* = 298.18
Orthorhombic, 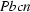

*a* = 23.534 (2) Å
*b* = 9.3318 (8) Å
*c* = 10.5047 (9) Å
*V* = 2307.0 (3) Å^3^
*Z* = 8
Mo *K*α radiation
μ = 0.16 mm^−1^
*T* = 298 K
0.30 × 0.20 × 0.10 mm

#### Data collection

Bruker SMART APEX CCD area-detector diffractometer
Absorption correction: none
16332 measured reflections
2855 independent reflections
2154 reflections with *I* > 2σ(*I*)
*R*_int_ = 0.033

#### Refinement

*R*[*F* ^2^ > 2σ(*F* ^2^)] = 0.046
*wR*(*F* ^2^) = 0.136
*S* = 1.06
2855 reflections
241 parameters
18 restraints
H-atom parameters constrained
Δρ_max_ = 0.22 e Å^−3^
Δρ_min_ = −0.26 e Å^−3^

### 

Data collection: *SMART* (Bruker, 2001 ▶); cell refinement: *SAINT-Plus* (Bruker, 2001 ▶); data reduction: *SAINT-Plus*; program(s) used to solve structure: *SHELXS97* (Sheldrick, 2008 ▶); program(s) used to refine structure: *SHELXL97* (Sheldrick, 2008 ▶); molecular graphics: *PLATON* (Spek, 2009 ▶); software used to prepare material for publication: *PLATON*.

## Supplementary Material

Crystal structure: contains datablocks global, I. DOI: 10.1107/S1600536809037416/bg2290sup1.cif

Structure factors: contains datablocks I. DOI: 10.1107/S1600536809037416/bg2290Isup2.hkl

Additional supplementary materials:  crystallographic information; 3D view; checkCIF report

## Figures and Tables

**Table 1:** Hydrogen-bond geometry (Å, °)

*D*—H⋯*A*	*D*—H	H⋯*A*	*D*⋯*A*	*D*—H⋯*A*
C3—H3⋯O2^i^	0.93	2.55	3.442 (10)	161
C9—H9⋯O5^ii^	0.93	2.53	3.286 (4)	139
N4—H4*A*⋯O6	0.86	2.44	3.2677 (16)	161
O1—H1*A*⋯O7	0.82	1.85	2.553 (2)	143

Symmetry codes: (i) 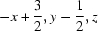; (ii) 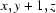.

**Fig. 1.**
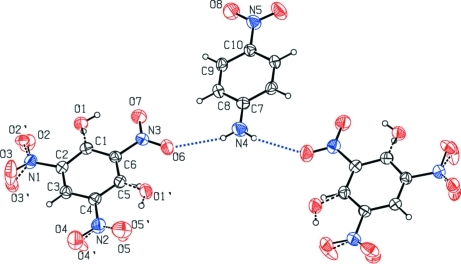
The title molecule with the atom-numbering scheme. The displacement ellipsoids are drawn at the 30% probability level. Hydrogen bonds are shown as blue dashed lines. Symmetry code a: 1-x, y, -z+1/2

**Fig. 2.**
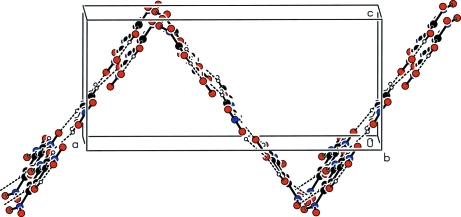
Section of the title structure, showing the two-dimensional (001) layer. Hydrogen bonds are shown as dashed lines. For the sake of clarity, the H atoms not involved in the hydrogen-bonds pattern have been omitted.

## supplementary crystallographic information

### Comment

Picric acid has been early used in the characterization of organic bases because of the ease of crystallization and hence purification when picrate derivatives were produced (Pascard *et al.*, 1982; Wang *et al.*, 2003; Pearson *et al.*, 2007; Harrison *et al.*, 2007). Here, we report the crystal structure of the title adduct, 2(C_6_H_3_N_3_O_7_).C_6_H_6_N_2_O_2_, (I), where the hydrogen atom was not transferred from the picric acid to the nitroaniline molecule, as expected, thus forming a neutral 1:2 molecular adduct (acid to base) (Fig.1). The 4-nitroaniline molecule is bisected by a mirror plane through the N4-C7···C10-N5 line, and the picric acid unit presents a number of disordered sites (see refinement section for details). In the latter acid group, the parameters of C1—O1 = 1.347 (4)Å and C6—C1—C2=115.2 (3)° are indicative of the proton presence, confirmed by the difference electron density map.

In the crystal packing, the molecular components are linked into a dimensional zigzag-like layer (Fig.2) running parallel to the (001) plane by a combination of intermolecular N—H···O, O—H···N and C—H···O hydrogen bonds (Table 1).

### Experimental

Picric acid (0.6873 g, 3 mmol) and 4-nitroaniline (0.4144 g, 3 mmol) were mixed in 10 ml e thanol.The mixture was kept at room temperature for two weeks,after which time needle like yellow crystals (0.40 *x* 0.08 *x* 0.03 mm) suitable for single-crystal X-ray diffraction were obtained.

### Refinement

In the refinement, the phenolic oxygen O1 and two sets of nitro-oxygen atoms ( O2/O3 and O4/O5) in the picric acid were found to be disordered over two positions. They were refined by using soft restraints (SHELXL commands PART, *DFIX* and SADI). The final occupancies refined to 0.81:0.19 (2), 0.55:0.45 (3) and 0.77:0.23 (4) for O1, O2/O3 and O4/O5 atoms, respectively.

Hydrogen H4A atom was first determined in the difference electron density map and placed at its idealized position with N—H = 0.86 Å and *U*_iso_(H) =1.2*U*_eq_(N). Owing to the disorder, hydrogen atoms attached to phenolic O1 or O1' atoms were placed also at the idealizeded positions with O—H = 0.82 Å and *U*_iso_(H) = 1.5*U*_eq_(N). The carbonic hydrogen atoms were positioned into their respective idealized positions, with C—H = 0.93 Å and *U*_iso_(H_aryl_) = 1.2*U*_eq_(C_aryl_).

### Crystal data

**Table:**

C_6_H_3_N_3_O_7_·0.5C_6_H_6_N_2_O_2_	*F*(000) = 1216
*M**_r_* = 298.18	*D*_x_ = 1.717 Mg m^−^^3^
Orthorhombic, *P**b**c**n*	Mo *K*α radiation, λ = 0.71073 Å
Hall symbol: -P 2n 2ab	Cell parameters from 5243 reflections
*a* = 23.534 (2) Å	θ = 2.4–27.1°
*b* = 9.3318 (8) Å	µ = 0.16 mm^−^^1^
*c* = 10.5047 (9) Å	*T* = 298 K
*V* = 2307.0 (3) Å^3^	Block, red
*Z* = 8	0.30 × 0.20 × 0.10 mm

### Data collection

**Table:**

Bruker SMART APEX CCD area-detector diffractometer	2154 reflections with *I* > 2σ(*I*)
Radiation source: fine focus sealed Siemens Mo tube	*R*_int_ = 0.033
graphite	θ_max_ = 28.3°, θ_min_ = 2.4°
0.3° wide ω exposures scans	*h* = −30→31
16332 measured reflections	*k* = −12→12
2855 independent reflections	*l* = −13→8

### Refinement

**Table:**

Refinement on *F*^2^	Secondary atom site location: difference Fourier map
Least-squares matrix: full	Hydrogen site location: inferred from neighbouring sites
*R*[*F*^2^ > 2σ(*F*^2^)] = 0.046	H-atom parameters constrained
*wR*(*F*^2^) = 0.136	*w* = 1/[σ^2^(*F*_o_^2^) + (0.0712*P*)^2^ + 0.2759*P*] where *P* = (*F*_o_^2^ + 2*F*_c_^2^)/3
*S* = 1.06	(Δ/σ)_max_ < 0.001
2855 reflections	Δρ_max_ = 0.22 e Å^−^^3^
241 parameters	Δρ_min_ = −0.26 e Å^−^^3^
18 restraints	Extinction correction: *SHELXL97* (Sheldrick, 2008), Fc^*^=kFc[1+0.001xFc^2^λ^3^/sin(2θ)]^-1/4^
Primary atom site location: structure-invariant direct methods	Extinction coefficient: 0.0032 (9)

### Special details

**Table:**

Geometry. All e.s.d.'s (except the e.s.d. in the dihedral angle between two l.s. planes) are estimated using the full covariance matrix. The cell e.s.d.'s are taken into account individually in the estimation of e.s.d.'s in distances, angles and torsion angles; correlations between e.s.d.'s in cell parameters are only used when they are defined by crystal symmetry. An approximate (isotropic) treatment of cell e.s.d.'s is used for estimating e.s.d.'s involving l.s. planes.
Refinement. Refinement of *F*^2^ against ALL reflections. The weighted *R*-factor *wR* and goodness of fit *S* are based on *F*^2^, conventional *R*-factors *R* are based on *F*, with *F* set to zero for negative *F*^2^. The threshold expression of *F*^2^ > σ(*F*^2^) is used only for calculating *R*-factors(gt) *etc*. and is not relevant to the choice of reflections for refinement. *R*-factors based on *F*^2^ are statistically about twice as large as those based on *F*, and *R*- factors based on ALL data will be even larger.

### Fractional atomic coordinates and isotropic or equivalent isotropic displacement parameters (Å^2^)

**Table:**

	*x*	*y*	*z*	*U*_iso_*/*U*_eq_	Occ. (<1)
C1	0.66852 (7)	0.40572 (18)	0.75606 (17)	0.0504 (4)	
H1	0.6733	0.5023	0.7753	0.061*	0.194 (3)
C2	0.69244 (6)	0.29633 (18)	0.83061 (16)	0.0496 (4)	
C3	0.68653 (7)	0.15487 (18)	0.80130 (17)	0.0517 (4)	
H3	0.7032	0.0852	0.8524	0.062*	
C4	0.65585 (7)	0.11617 (18)	0.69592 (17)	0.0511 (4)	
C5	0.63050 (7)	0.21640 (19)	0.61822 (16)	0.0508 (4)	
H5	0.6098	0.1890	0.5468	0.061*	0.806 (3)
C6	0.63686 (6)	0.35912 (17)	0.65015 (16)	0.0488 (4)	
C7	0.5000	0.6849 (3)	0.2500	0.0505 (5)	
C8	0.52386 (7)	0.76184 (19)	0.35141 (16)	0.0533 (4)	
H8	0.5397	0.7127	0.4198	0.064*	
C9	0.52404 (7)	0.90824 (19)	0.35084 (15)	0.0533 (4)	
H9	0.5403	0.9585	0.4181	0.064*	
C10	0.5000	0.9812 (3)	0.2500	0.0515 (6)	
N4	0.5000	0.5397 (3)	0.2500	0.0760 (7)	
H4A	0.5148	0.4936	0.3127	0.091*	
N5	0.5000	1.1374 (3)	0.2500	0.0705 (6)	
N1	0.72518 (7)	0.33052 (17)	0.94650 (15)	0.0607 (4)	
N2	0.64972 (8)	−0.03633 (18)	0.66686 (19)	0.0733 (5)	
N3	0.60883 (7)	0.46447 (16)	0.56958 (16)	0.0598 (4)	
O1	0.67753 (7)	0.54438 (16)	0.78695 (17)	0.0670 (6)	0.81 (2)
H1A	0.6603	0.5962	0.7370	0.080*	0.806 (3)
O2	0.7225 (5)	0.4511 (5)	0.9832 (7)	0.084 (2)	0.55 (3)
O3	0.7584 (9)	0.2434 (12)	0.980 (2)	0.130 (5)	0.55 (3)
O5	0.6212 (4)	−0.0676 (7)	0.5744 (6)	0.100 (2)	0.77 (4)
O4	0.6739 (6)	−0.1218 (7)	0.7350 (9)	0.108 (2)	0.77 (4)
O1'	0.6057 (3)	0.1603 (7)	0.5099 (6)	0.079 (3)	0.19 (2)
H1'	0.6101	0.0731	0.5088	0.119*	0.194 (3)
O2'	0.7386 (8)	0.4462 (8)	0.9798 (8)	0.093 (3)	0.45 (3)
O3'	0.7319 (7)	0.2298 (7)	1.0257 (8)	0.082 (3)	0.45 (3)
O4'	0.6609 (16)	−0.120 (3)	0.752 (2)	0.106 (8)	0.23 (4)
O5'	0.6331 (17)	−0.077 (3)	0.5624 (14)	0.108 (8)	0.23 (4)
O6	0.58058 (7)	0.42228 (16)	0.48088 (16)	0.0820 (5)	
O7	0.61414 (7)	0.59241 (15)	0.59443 (18)	0.0839 (5)	
O8	0.52462 (9)	1.20034 (17)	0.33516 (17)	0.1041 (7)	

### Atomic displacement parameters (Å^2^)

**Table:**

	*U*^11^	*U*^22^	*U*^33^	*U*^12^	*U*^13^	*U*^23^
C1	0.0482 (8)	0.0479 (9)	0.0552 (10)	−0.0025 (6)	0.0033 (7)	−0.0010 (7)
C2	0.0454 (8)	0.0525 (9)	0.0508 (9)	−0.0017 (7)	−0.0013 (7)	−0.0044 (7)
C3	0.0508 (8)	0.0497 (9)	0.0545 (10)	0.0053 (7)	−0.0042 (7)	−0.0011 (7)
C4	0.0519 (9)	0.0474 (9)	0.0541 (10)	0.0002 (7)	−0.0010 (7)	−0.0065 (7)
C5	0.0465 (8)	0.0576 (10)	0.0483 (9)	−0.0035 (7)	−0.0009 (7)	−0.0014 (8)
C6	0.0445 (8)	0.0505 (9)	0.0515 (10)	−0.0010 (7)	0.0029 (7)	0.0063 (7)
C7	0.0487 (11)	0.0499 (12)	0.0531 (13)	0.000	0.0063 (10)	0.000
C8	0.0554 (9)	0.0604 (10)	0.0441 (9)	0.0068 (7)	−0.0050 (7)	0.0052 (7)
C9	0.0587 (10)	0.0594 (10)	0.0417 (9)	0.0001 (7)	−0.0079 (7)	−0.0046 (7)
C10	0.0584 (13)	0.0478 (12)	0.0482 (13)	0.000	−0.0035 (10)	0.000
N4	0.1002 (18)	0.0528 (13)	0.0750 (17)	0.000	−0.0071 (14)	0.000
N5	0.0951 (17)	0.0549 (13)	0.0614 (14)	0.000	−0.0084 (13)	0.000
N1	0.0598 (9)	0.0596 (9)	0.0626 (10)	−0.0030 (7)	−0.0093 (7)	−0.0083 (8)
N2	0.0863 (12)	0.0529 (9)	0.0805 (13)	0.0035 (9)	−0.0180 (10)	−0.0131 (9)
N3	0.0598 (9)	0.0540 (9)	0.0657 (10)	−0.0041 (7)	−0.0051 (8)	0.0115 (7)
O1	0.0826 (12)	0.0428 (8)	0.0757 (12)	−0.0040 (7)	−0.0190 (9)	−0.0021 (7)
O2	0.103 (4)	0.060 (2)	0.090 (3)	0.002 (3)	−0.024 (2)	−0.039 (3)
O3	0.137 (8)	0.103 (4)	0.149 (9)	0.046 (4)	−0.097 (7)	−0.042 (4)
O5	0.110 (3)	0.066 (2)	0.124 (5)	0.015 (2)	−0.059 (3)	−0.038 (3)
O4	0.151 (4)	0.047 (2)	0.127 (5)	0.0128 (19)	−0.064 (4)	−0.011 (3)
O1'	0.093 (5)	0.075 (5)	0.070 (5)	−0.001 (4)	−0.025 (4)	0.002 (4)
O2'	0.123 (7)	0.084 (4)	0.073 (3)	−0.062 (4)	−0.037 (3)	0.030 (4)
O3'	0.110 (6)	0.058 (3)	0.078 (4)	0.012 (3)	−0.040 (3)	−0.002 (2)
O4'	0.20 (2)	0.045 (7)	0.068 (8)	0.000 (9)	0.011 (13)	0.001 (5)
O5'	0.174 (18)	0.098 (13)	0.052 (8)	−0.035 (11)	0.004 (10)	−0.026 (7)
O6	0.1017 (11)	0.0681 (9)	0.0763 (10)	−0.0034 (8)	−0.0344 (9)	0.0128 (7)
O7	0.0925 (10)	0.0513 (8)	0.1079 (12)	−0.0020 (7)	−0.0244 (9)	0.0104 (8)
O8	0.1598 (18)	0.0609 (9)	0.0916 (12)	−0.0151 (10)	−0.0387 (11)	−0.0121 (9)

### Geometric parameters (Å, °)

**Table:**

C1—O1	1.351 (2)	C9—C10	1.381 (2)
C1—C2	1.404 (2)	C9—H9	0.9300
C1—C6	1.408 (2)	C10—C9^i^	1.381 (2)
C1—H1	0.9300	C10—N5	1.457 (3)
C2—C3	1.363 (2)	N4—H4A	0.8600
C2—N1	1.476 (2)	N5—O8^i^	1.2169 (18)
C3—C4	1.370 (2)	N5—O8	1.2169 (18)
C3—H3	0.9300	N1—O2'	1.178 (6)
C4—C5	1.377 (2)	N1—O3	1.182 (5)
C4—N2	1.463 (2)	N1—O2	1.191 (5)
C5—C6	1.382 (2)	N1—O3'	1.265 (5)
C5—O1'	1.382 (6)	N2—O4	1.214 (5)
C5—H5	0.9300	N2—O5	1.216 (4)
C6—N3	1.455 (2)	N2—O4'	1.221 (10)
C7—N4	1.355 (3)	N2—O5'	1.225 (10)
C7—C8	1.402 (2)	N3—O6	1.210 (2)
C7—C8^i^	1.402 (2)	N3—O7	1.228 (2)
C8—C9	1.366 (2)	O1—H1A	0.8200
C8—H8	0.9300	O1'—H1'	0.8200
			
O1—C1—C2	119.97 (16)	C10—C9—H9	120.2
O1—C1—C6	124.67 (16)	C9—C10—C9^i^	120.9 (2)
C2—C1—C6	115.35 (15)	C9—C10—N5	119.56 (11)
C2—C1—H1	122.3	C9^i^—C10—N5	119.56 (11)
C6—C1—H1	122.3	C7—N4—H4A	120.0
C3—C2—C1	122.50 (16)	O8^i^—N5—O8	122.3 (3)
C3—C2—N1	116.69 (15)	O8^i^—N5—C10	118.85 (13)
C1—C2—N1	120.81 (15)	O8—N5—C10	118.85 (13)
C2—C3—C4	119.45 (16)	O2'—N1—O3	111.4 (6)
C2—C3—H3	120.3	O3—N1—O2	126.1 (5)
C4—C3—H3	120.3	O2'—N1—O3'	116.8 (5)
C3—C4—C5	121.88 (16)	O2—N1—O3'	119.7 (6)
C3—C4—N2	118.49 (16)	O2'—N1—C2	125.7 (5)
C5—C4—N2	119.63 (16)	O3—N1—C2	116.3 (4)
C4—C5—C6	117.63 (16)	O2—N1—C2	116.3 (4)
C4—C5—O1'	114.4 (3)	O3'—N1—C2	116.6 (4)
C6—C5—O1'	127.7 (3)	O4—N2—O5	124.9 (4)
C4—C5—H5	121.2	O5—N2—O4'	123.6 (15)
C6—C5—H5	121.2	O4—N2—O5'	118.4 (16)
C5—C6—C1	123.16 (15)	O4'—N2—O5'	122.1 (15)
C5—C6—N3	117.44 (15)	O4—N2—C4	118.0 (4)
C1—C6—N3	119.39 (15)	O5—N2—C4	117.0 (3)
N4—C7—C8	120.81 (11)	O4'—N2—C4	116.7 (13)
N4—C7—C8^i^	120.81 (11)	O5'—N2—C4	121.3 (13)
C8—C7—C8^i^	118.4 (2)	O6—N3—O7	122.38 (16)
C9—C8—C7	120.67 (16)	O6—N3—C6	118.48 (15)
C9—C8—H8	119.7	O7—N3—C6	119.13 (16)
C7—C8—H8	119.7	C1—O1—H1A	109.5
C8—C9—C10	119.70 (16)	C5—O1'—H1'	109.5
C8—C9—H9	120.2		
			
O1—C1—C2—C3	−177.52 (17)	C9—C10—N5—O8^i^	−175.18 (14)
C6—C1—C2—C3	1.3 (2)	C9^i^—C10—N5—O8^i^	4.82 (14)
O1—C1—C2—N1	3.0 (2)	C9—C10—N5—O8	4.82 (14)
C6—C1—C2—N1	−178.16 (14)	C9^i^—C10—N5—O8	−175.18 (14)
C1—C2—C3—C4	−0.5 (3)	C3—C2—N1—O2'	173.0 (10)
N1—C2—C3—C4	179.01 (15)	C1—C2—N1—O2'	−7.5 (10)
C2—C3—C4—C5	−0.1 (3)	C3—C2—N1—O3	24.2 (17)
C2—C3—C4—N2	−179.55 (16)	C1—C2—N1—O3	−156.3 (17)
C3—C4—C5—C6	−0.1 (3)	C3—C2—N1—O2	−168.1 (6)
N2—C4—C5—C6	179.26 (16)	C1—C2—N1—O2	11.4 (6)
C3—C4—C5—O1'	174.3 (4)	C3—C2—N1—O3'	−18.2 (9)
N2—C4—C5—O1'	−6.3 (5)	C1—C2—N1—O3'	161.3 (9)
C4—C5—C6—C1	1.1 (2)	C3—C4—N2—O4	−2.7 (9)
O1'—C5—C6—C1	−172.5 (5)	C5—C4—N2—O4	177.9 (9)
C4—C5—C6—N3	−178.51 (15)	C3—C4—N2—O5	178.4 (6)
O1'—C5—C6—N3	7.9 (5)	C5—C4—N2—O5	−1.0 (6)
O1—C1—C6—C5	177.14 (16)	C3—C4—N2—O4'	16 (2)
C2—C1—C6—C5	−1.6 (2)	C5—C4—N2—O4'	−163 (2)
O1—C1—C6—N3	−3.3 (3)	C3—C4—N2—O5'	−165 (2)
C2—C1—C6—N3	177.95 (14)	C5—C4—N2—O5'	15 (2)
N4—C7—C8—C9	179.68 (12)	C5—C6—N3—O6	1.5 (2)
C8^i^—C7—C8—C9	−0.32 (12)	C1—C6—N3—O6	−178.11 (17)
C7—C8—C9—C10	0.6 (2)	C5—C6—N3—O7	−179.18 (17)
C8—C9—C10—C9^i^	−0.31 (12)	C1—C6—N3—O7	1.2 (2)
C8—C9—C10—N5	179.68 (12)		

Symmetry codes: (i) −*x*+1, *y*, −*z*+1/2.

### Hydrogen-bond geometry (Å, °)

**Table:**

*D*—H···*A*	*D*—H	H···*A*	*D*···*A*	*D*—H···*A*
C3—H3···O2^ii^	0.93	2.55	3.442 (10)	161
C9—H9···O5^iii^	0.93	2.53	3.286 (4)	139
N4—H4A···O6	0.86	2.44	3.2677 (16)	161
O1—H1A···O7	0.82	1.85	2.553 (2)	143

Symmetry codes: (ii) −*x*+3/2, *y*−1/2, *z*; (iii) *x*, *y*+1, *z*.
